# PLK1 and PARP positively correlate in Middle Eastern breast cancer and their combined inhibition overcomes PARP inhibitor resistance in triple negative breast cancer

**DOI:** 10.3389/fonc.2023.1286585

**Published:** 2024-01-03

**Authors:** Abdul K. Siraj, Pratheesh Kumar Poyil, Divya Padmaja, Sandeep Kumar Parvathareddy, Khadija Alobaisi, Saravanan Thangavel, Roxanne Diaz, Rafia Begum, Osama Almalik, Fouad Al-Dayel, Khawla S. Al-Kuraya

**Affiliations:** ^1^ Human Cancer Genomic Research, King Faisal Specialist Hospital and Research Center, Riyadh, Saudi Arabia; ^2^ Department of Surgery, King Faisal Specialist Hospital and Research Center, Riyadh, Saudi Arabia; ^3^ Department of Pathology, King Faisal Specialist Hospital and Research Centre, Riyadh, Saudi Arabia

**Keywords:** PLK1, PARP, TNBC, apoptosis, stemness

## Abstract

**Background:**

Despite advancements in treatment approaches, patients diagnosed with aggressive breast cancer (BC) subtypes typically face an unfavorable prognosis. Globally, these cancers continue to pose a significant threat to women's health, leading to substantial morbidity and mortality. Consequently, there has been a significant struggle to identify viable molecular targets for therapeutic intervention in these patients. Polo-like Kinase-1 (PLK1) represents one of these molecular targets currently undergoing rigorous scrutiny for the treatment of such tumors. Yet, its role in the pathogenesis of BC in Middle Eastern ethnicity remains unexplored.

**Methods:**

We investigated the expression of PLK1 protein in a cohort of more than 1500 Middle Eastern ethnicity BC cases by immunohistochemistry. Association with clinicopathological parameters and prognosis were performed. *In vitro* studies were conducted using the PLK1 inhibitor volasertib and the PARP inhibitor olaparib, either alone or in combination, in PTC cell lines.

**Results:**

Overexpression of PLK1 was detected in 27.4% of all BC cases, and this was notably correlated with aggressive clinicopathological markers. PLK1 was enriched in the triple-negative breast cancer (TNBC) subtype and exhibited poor overall survival (p = 0.0347). Notably, there was a positive correlation between PLK1 and PARP overexpression, with co-expression of PLK1 and PARP observed in 15.7% of cases and was associated with significantly poorer overall survival (OS) compared to the overexpression of either protein alone (p = 0.0050). *In vitro*, we studied the effect of PLK1 and PARP inhibitors either single or combined treatments in two *BRCA* mutated, and one *BRCA* proficient TNBC cell lines. We showed that combined inhibition significantly reduced cell survival and persuaded apoptosis in TNBC cell lines. Moreover, our findings indicate that inhibition of PLK1 can reinstate sensitivity in PARP inhibitor (PARPi) resistant TNBC cell lines.

**Conclusion:**

Our results shed light on the role of PLK1 in the pathogenesis and prognosis of Middle Eastern BC and support the potential clinical development of combined inhibition of PLK1 and PARP, a strategy that could potentially broaden the use of PLK1 and PARP inhibitors beyond BC cases lacking *BRCA*.

## Graphical Abstract

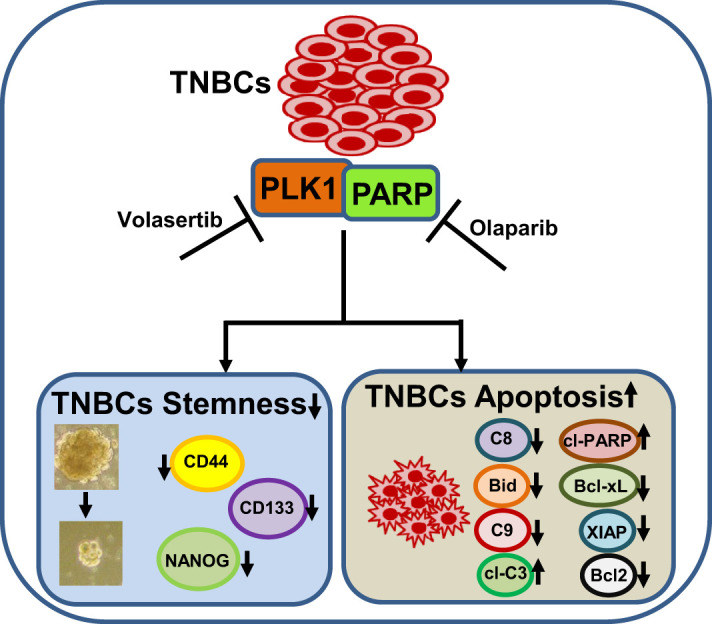

## Introduction

Breast cancer (BC) remains one of the deadliest diseases globally, posing a substantial menace to women’s well-being (1, 2). Among its subtypes, triple-negative breast cancer (TNBC), distinguished by the absence of estrogen receptors (ER), progesterone receptors (PR), and human epidermal growth factor receptor 2 (HER2) expression, denotes one of the most aggressive forms of BC and is marked by rapid recurrence, early metastasis, and a bleak prognosis (3–5). Unfortunately, a significant proportion of TNBC patients continue to experience early relapse and distant metastasis due to the limited efficacy of existing treatments (6, 7). Consequently, there is an urgent demand for the identification of molecular therapeutic targets and strategies to combat aggressive BC.

The considerable heterogeneity and the scarceness of genetic targets in TNBC have restricted therapeutic advancements over the past few decades (8, 9). It is worth noting that approximately 20-30% of TNBC cases exhibit confirmed *BRCA1/2* mutations (10–12). Furthermore, the molecular markers linked to “*BRCA*ness” significantly expand the group of individuals with *BRCA* mutations and deficiencies (13). Consequently, it is assumed that TNBC patients with *BRCA* mutations should be more responsive to DNA damage treatments. Nevertheless, the clinical outcomes have not met these expectations (14).

Tumor cells harboring *BRCA1/2* mutations exhibit a deficiency in homologous recombination (HR)-mediated DNA repair, making them particularly susceptible to treatment with poly(adenosine diphosphate-ribose) polymerase (PARP) inhibitors (15, 16). PARP plays a vital role in the repair of single-strand breaks (SSBs). Upon detection of SSBs, PARP initiates the base excision repair pathway to rectify these abnormalities (17, 18). Furthermore, in cases involving double-strand breaks (DSBs), PARP is involved in the repair process through either homologous recombination (HR) or the non-homologous end joining pathways (19).

PARP inhibitors (PARPi) have demonstrated approved effectiveness in various cancer types, including breast cancer, particularly in HR-deficient tumors (20, 21). Conversely, their effectiveness is constrained in HR-proficient tumors (22). Nevertheless, PARPi resistance is inevitable (23, 24). Consequently, it is imperative to identify combination therapies that can enhance the sensitivity of tumor cells to PARPi and potentially overcome PARPi resistance, especially in TNBC, to expand the benefit of these therapies. Furthermore, we recently unveiled that PARP overexpression serves as an independent prognostic marker for Middle Eastern BC patients (25).

The serine/threonine polo-like kinase-1 (PLK1) plays crucial roles in the regulation of cell division. PLK1 oversees the control of cytokinesis, orchestrates mitotic entry, facilitates spindle assembly, and regulates chromosome dynamics (26–30). Additionally, PLK1 intricately participates in overseeing the control of DNA damage repair by exerting control over various critical repair proteins within the homologous recombination (HR) pathway (31, 32). Of notable significance, PLK1 activity is requisite for cells to re-enter the cell cycle following recovery from DNA damage-induced G2 arrest (33, 34). Dysregulation of PLK1 has been documented in various tumor types, contributing significantly to tumor development and progression (35, 36). Consequently, PLK1 inhibitors have been developed and are currently under examination as potential anti-cancer agents (37, 38).

High PLK1 expression is a common feature in numerous cancer types (39–45), and elevated PLK1 levels have been linked to aggressive tumor traits, including TNBC, vascular invasion, high proliferation rates, and poor prognostic implications in BC (46, 47). Nevertheless, the role of PLK1 in BC, particularly in understudied ethnicities such as Middle Eastern BC remains unexplored.

In this research, our initial focus was on evaluating the expression of PLK1 protein in a large cohort of Middle Eastern BC cases, examining its relationship with clinico-pathological factors and survival outcomes. Subsequently, we conducted *in vitro* analyses to assess the effect of combining PLK1 and PARP inhibitors. Moreover, our investigation revealed that PLK1 inhibition effectively reverses PARP inhibitor resistance in TNBC cell lines, irrespective of their *BRCA1/2* mutation status. These findings unequivocally establish the significant role of PLK1 in the pathogenesis of Middle Eastern BC and underscore the potential for combined PLK1 and PARP inhibitor therapy to extend its use beyond BC cases lacking *BRCA*.

## Materials and methods

### Patient selection and clinico-pathological data

The study encompassed a total of 1,536 breast cancer patients who were diagnosed between 1989 and 2018 at the King Faisal Specialist Hospital and Research Centre in Riyadh, Saudi Arabia. Baseline clinico-pathological data were retrieved from case records and are summarized in **Table 1**. We classified the histologic subtype of each breast tumor sample according to the 2019 World Health Organization (WHO) classification of breast tumors. The staging of BC was carried out in compliance with the eighth edition of the American Joint Committee on Cancer (AJCC) staging system (48). Overall survival (OS) was defined as the duration from the date of diagnosis to the point at which patients diagnosed with the disease were still alive.

**Table 1:** Clinico-pathological variables for the patient cohort (n = 1536).

Clinico-pathologic variables	n (%)
Age (years)	
Median (range)	45.3 (13.0 – 94.0)
≤50	1035 (67.4)
>50	501 (32.6)
Histological subtype	
Infiltrating Ductal carcinoma	1415 (92.1)
Infiltrating Lobular carcinoma	100 (6.5)
Mucinous carcinoma	21 (1.4)
pT	
T1	401 (26.1)
T2	697 (45.4)
T3	196 (12.8)
T4	157 (10.2)
Unknown	85 (5.5)
pN	
N0	505 (32.9)
N1-N3	873 (56.8)
Unknown	158 (10.3)
pM	
M0	1363 (88.8)
M1	125 (8.1)
Unknown	48 (3.1)
Tumor Stage	
I	194 (12.6)
II	639 (41.7)
III	481 (31.3)
IV	125 (8.1)
Unknown	97 (6.3)
Histologic Grade	
Well differentiated	120 (7.8)
Moderately differentiated	746 (48.6)
Poorly differentiated	604 (39.3)
Unknown	66 (4.3)
Estrogen Receptor	
Positive	999 (65.0)
Negative	481 (31.3)
Unknown	56 (3.7)
Progesterone Receptor	
Positive	886 (57.7)
Negative	592 (38.5)
Unknown	58 (3.8)
Her-2 neu	
Positive	376 (24.5)
Negative	1085 (70.6)
Unknown	75 (4.9)
Molecular subtype	
Luminal	1050 (68.4)
Her-2 positive	183 (11.9)
Triple negative	230 (15.0)
Unknown	73 (4.7)

### Ethics declarations

Ethical approval for the present study was granted by the Institutional Review Board of the King Faisal Specialist Hospital and Research Centre. Additionally, the Research Advisory Council (RAC) issued a waiver of informed consent for the utilization of archival tissue specimens and retrospective patient case data under project RAC# 2220 013. All methods employed in this study adhered to the principles outlined in the Declaration of Helsinki.

### Tissue microarray (TMA) construction and immunohistochemistry (IHC) analysis

Tissue Microarray (TMA) format was utilized for immunohistochemical analysis of the BC samples. TMA was constructed following established procedures (49). In brief, we utilized a modified semiautomatic robotic precision instrument (Beecher Instruments, Woodland, WI) to extract tissue cylinders with a diameter of 0.6 mm from representative tumor regions of the donor tissue block. These tissue cylinders were then integrated into the recipient paraffin block. For each case, two cores of BC tissue were arrayed.

TMA slides were manually processed and stained in accordance with previously established protocols (50). Primary antibodies against PLK1 (mouse monoclonal, ab-17056, 1:500, pH 9.0; Abcam, Cambridge, United Kingdom) and PARP (clone F-2, 1:300, pH 6.0; Santa Cruz Biotechnology, CA, USA). The Dako Envision Plus System kit was used as the secondary detection system with 3, 30-diaminobenzidine as chromogen. All slides were counter stained with hematoxylin, dehydrated, cleared and mounted. Negative controls included omission of the primary antibody. Additionally, TMA included normal tissues from various organ systems to serve as control. To minimize the potential effects of slide aging and enhance the reproducibility of the experiment, only freshly cut slides were stained simultaneously.

PLK1 staining was scored using immunoreactivity score (IRS), as described previously (51). Briefly, staining intensity was scored as 0: negative; 1: weak; 2: moderate; or 3: strong, and staining proportion was scored as 0, 0%; 1, 1–10%; 2, 11–50%; 3, 51–80%; or 4, more than 80% positive cells. The final IRS score was calculated by multiplying the intensity and proportion scores. Low expression of PLK1 was defined as IRS 0–6 and high expression of PLK1 was defined as IRS more than 6. PARP staining was scored using the quick score (QS) method, as described previously (25). Based on the QS, nuclear PARP expression was graded as low (0–9) or high (10–18). The cut-off for high Ki67 was taken as more than 10% nuclear staining (52).

### Cell culture

The cell lines, MCF-10A, MCF-7, CAL-120, CAL-51, EFM-19, EVSAT, MDA-MB-231, MT3 and HDQP1 were procured from ATCC (American Type Culture Collection) and cultured in RPMI 1640 medium. Media were supplemented with 10% fetal bovine serum (FBS), 1% penicillin/streptomycin and cells were grown at 37°C in a humidified (95%) CO_2_ (5%) incubator.

### Reagents and antibodies

The PARP inhibitor, olaparib and PLK1 inhibitor, volasertib were obtaind from Selleck Chemicals (Houston, TX, USA). Antibodies against PLK1 (4513), Bcl-xl (2762), Bcl-2 (2876), cleaved caspase-3 (9664), PARP (9542), CD133 (64326), CD44 (3570), NANOG (4903) and GAPDH (5174) were procured from Cell Signaling Technology (Danvers, MA). Antibodies against Caspase-9 (sc-17784) and caspase-3 (sc-56053) were obtained from Santa Cruz Biotechnology, Inc. (Santa Cruz, CA). Caspase-8 (51-8084) and XIAP (610763) antibodies were procured from BD Pharmingen (San Diego, CA, USA). Annexin V was obtained from Thermo Fischer Scientific (Waltham, MA).

### Cell viability assay

The cell viability was assessed using MTT assay (25). Briefly, TNBC cells were seeded into a 96-well plate, and they were treated to various concentrations of olaparib and volasertib, either individually or in combination, for a 48-hour period. Before 4 h completion of incubation, 10 µl MTT (5 mg/ml) was added. The cultures were solubilized and spectrophotometric absorbance was detected at 490 nm with a VersaMAx microplate reader (Molecular Devices, San Jose, CA, USA). The relative cell viability (%) was expressed as a percentage relative to the untreated control cells (n=6).

### Annexin V staining

The apoptosis assay was conducted following established protocols (25). Briefly, TNBC cells were exposed to various concentrations of olaparib and volasertib for a duration of 48 hours. Subsequently, the cells were collected, stained with annexin V-FITC/PI, and data were analyzed using FACSCalibur flow cytometer (BD Biosciences, NJ, USA) and CellQuest software (BD Biosciences).

### Cell lysis and western blotting

TNBC cells after indicated treatment, cells were lysed in phosphorylation lysis buffer as previously described (25). Protein concentrations were measured using the Bio-Rad assay system. Equal amounts of proteins (5–10 μg) were separated by SDS–PAGE (sodium dodecyl sulfate-polyacrylamide gel electrophoresis) and then transferred to a polyvinylidene difluoride (PVDF) membrane (Immobilon, Millipore, Billerica, MA, USA). The membranes were immunoblotted using primary antibodies against PLK1 (1:1000), Bcl-xl (1:1000), Bcl-2 (1:1000), cleaved caspase-3 (1:1000), PARP (1:2000), CD133 (1:1000), CD44 (1:2000), NANOG (1:1000), Caspase-9 (1:1000), caspase-3 (1:2000), Caspase-8 (1:2000), XIAP (1:2000) and GAPDH (1:2000). The signals from the primary antibody were amplified by incubating with horse radish peroxidase conjugated anti-rabbit/mouse IgG (1:5000; Cell Signaling Technology) and visualized through the enhanced chemiluminescence (Amersham, Piscataway, NJ, USA) method.

### Transfection

The knockdown of PLK1 in TNBC cell lines were carried out using Lipofectamine™2000 (Invitrogen, Carlsbad, CA) in accordance with manufacturer’s recommendation. Briefly, cells were seeded into 6-well culture plates and transfected with two different sequence of pRS-PLK1 shRNA’s (TR320457A and TR320457B) and scrambled pRS-shRNA control (TR30012) from Origene (Rockville, MD) for 48 hours. Stable PLK1 knockdown clones were isolated by puromycin selection and the knockdown of PLK1 protein was confirmed by immunoblotting.

### Sphere forming assay

The sphere forming assay was performed as described earlier (53). The TNBC cells (500/well) were seeded into Corning 24-well ultra-low attachment plates cultured in spheroid culture medium (DMEM-F12, B27, EGF (20 ng/ml), BSA (0.4%) and insulin (4 μg/ml). The cultures were supplemented with fresh growth media every 48 hours. At day 7, the number of successful spheroids formed were counted and photographed under an Olympus CKX41 microscope with the cellSens Entry software.

### Statistical analysis

We used contingency table analysis and Chi-square tests to investigate the associations between clinico-pathological variables and protein expression. Mantel-Cox log-rank test was used to evaluate overall survival. We generated survival curves using the Kaplan-Meier method. For statistical analyses, we employed two-sided tests with a significance threshold set at a p-value of < 0.05. Data analyses was done using the JMP14.0 (SAS Institute, Inc., Cary, NC) software package.

For all functional studies, data presented are means ± SD of triplicates in an independent experiment, which was repeated for at least two times with the same results. For multiple comparisons, one-way analysis of variance (ANOVA) was performed using IBM SPSS Statistics 21 software (IBM Corp., Armonk, NY). Values of p < 0.05 were considered statistically significant.

## Results

### Patient characteristics

Median age of the study population was 45.3 years (range: 13 – 94 years). Infiltrating ductal carcinoma was the most common histologic subtype, accounting for 92.1% (1415/1536) of BC. Majority of the patients had moderately to poorly differentiated tumors (87.9%, 1350/1536) with 8.1% (125/1536) presenting with distant metastasis at diagnosis. 65.0% (999/1536) of tumors were ER positive, 57.7% (886/1536) were progesterone receptor (PR) positive and 24.5% (376/1536) were Her-2 neu positive. 15.0% (230/1536) of tumors were triple negative breast cancers (**Table 1**).

### The expression of PLK1 in BC and its associations with clinico-pathological factors

**Figure 1:**
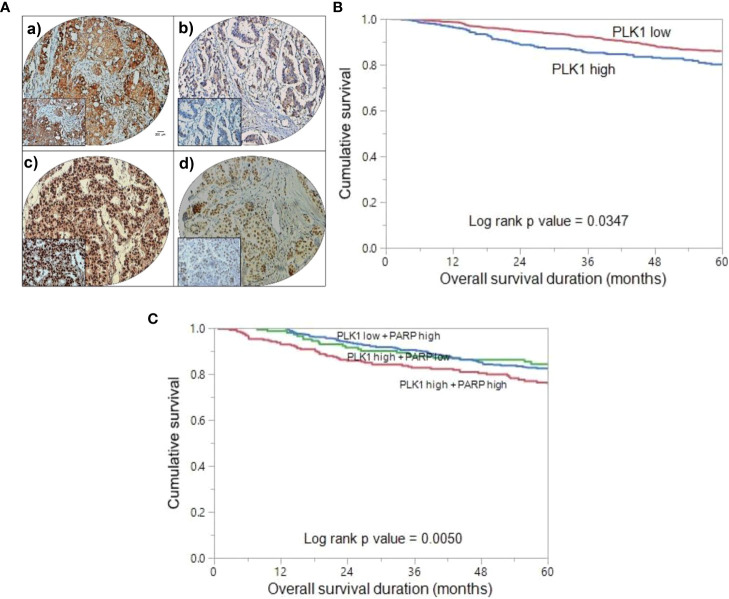
Tissue microarray (TMA) based immunohistochemistry analysis of PLK1 and PARP in BC patients and overall survival (OS) analysis. **(A)** BC TMA spots showing PLK1 (a) and PARP (c) overexpression. Conversely, a separate group of TMA spots exhibits diminished expression of PLK1 (b) and PARP (d). 20 X/0.70 objective on an Olympus BX 51 microscope (Olympus America Inc, Center Valley, PA, USA) with the inset showing a 40X 0.85 aperture magnified view of the same TMA spot. (Scale bar=200 µm) **(B)** Kaplan Meier survival plot showing significant difference in poor OS between PLK1 high expression and PLK1 low expression cases (p = 0.0347). **(C)** Kaplan Meier survival plot showing statistically significant poor OS in BC patients who co-express PLK1 and PARP, compared to over-expression of either of the two proteins alone (p = 0.0050).

Immunohistochemical analysis for PLK1 protein was performed in 1536 BC samples. However, the data could be accurately interpreted in 1494 of the samples and these samples were included for subsequent analysis. PLK1 over-expression was observed in 27.4% (410/1494) of cases (**Figure 1A**), and this was found to be significantly correlated with adverse clinico-pathological parameters such as older age (p = 0.0046), poorly differentiated tumors (p < 0.0001), ER negative (p < 0.0001), PR negative (p = 0.0017), triple negative breast cancer (p < 0.0001) and high proliferative index (Ki-67; p < 0.0001) (**Table 2**). Interestingly, we also found a significant association between PLK1 expression and PARP over-expression (p < 0.0001) in our cohort (**Table 2**).

**Table 2:** Correlation of PLK1 protein expression with clinico-pathological parameters in breast cancer.

	Total		PLK1 High		PLK1 Low		p value
	N	%	N	%	N	%	
**Total Number of Cases**	1494		410	27.4	1084	72.6	
Age Groups (years)							
≤ 50	1009	67.5	254	61.9	755	69.6	0.0046
>50	485	32.5	156	38.1	329	30.4	
Histological subtype							
Infiltrating Ductal Carcinoma	1382	92.5	392	95.6	990	91.3	0.0178
Infiltrating Lobular Carcinoma	92	6.2	14	3.4	78	7.2	
Mucinous Carcinoma	20	1.3	4	1.0	16	1.5	
Histological Grade							
Well differentiated	115	8.0	16	4.0	99	9.7	< 0.0001
Moderately differentiated	720	50.3	169	41.7	551	53.7	
Poorly differentiated	596	41.7	220	54.3	376	36.6	
pT							
T1	388	27.5	102	25.7	286	28.2	0.7413
T2	680	48.2	196	49.5	484	47.7	
T3	187	13.3	51	12.9	136	13.4	
T4	155	11.0	47	11.9	108	10.7	
pN							
N0	489	36.5	147	39.3	342	35.4	0.1833
N1-N3	851	63.5	227	60.7	624	64.6	
pM							
M0	1325	91.5	368	90.4	957	91.9	0.3532
M1	123	8.5	39	9.6	84	8.1	
Tumor Stage							
I	189	13.5	60	15.0	129	12.9	0.3362
II	618	44.2	180	45.0	438	43.9	
III	469	33.5	121	30.2	348	34.8	
IV	123	8.8	39	9.8	84	8.4	
Estrogen receptor							
Positive	965	67.0	240	59.3	725	70.0	< 0.0001
Negative	475	33.0	165	40.7	310	30.0	
Progesterone receptor							
Positive	860	59.8	216	53.3	644	62.3	0.0017
Negative	578	40.2	189	46.7	389	37.7	
Her-2 neu							
Positive	365	25.7	104	25.7	261	25.7	0.9755
Negative	1056	74.3	300	74.3	756	74.3	
Molecular subtype							
Luminal	1016	71.4	258	63.9	758	74.4	< 0.0001
Her-2 positive	181	12.7	47	11.6	134	13.1	
Triple negative	226	15.9	99	24.5	127	12.5	
Ki-67							
High (>10%)	875	59.9	298	74.1	577	54.5	< 0.0001
Low (≤10%)	585	40.1	104	25.9	481	45.5	
PARP IHC							
High	696	47.4	235	58.2	461	43.3	< 0.0001
Low	772	52.6	169	41.8	603	56.7	

### PLK1 expression and clinical outcome

In our BC cohort, we conducted an analysis to investigate the relationship between PLK1 over-expression and clinical outcomes. Kaplan Meier curve analysis revealed that patients exhibiting PLK-1 over-expression had a significantly worse OS compared to those who had low PLK-1 expression (p = 0.0347) (**Figure 1B**). Next, we aimed to assess the prognostic significance of co-expression of PLK1 and PARP. We found that tumors exhibiting co-expression of PLK1 and PARP had a significantly worse OS compared to those who had either PLK1 over-expression alone or PARP over-expression alone (p = 0.0050) (**Figure 1C**).

### Inhibition of PLK1 hinders TNBC cell growth *in vitro* and *in vivo*

**Figure 2:**
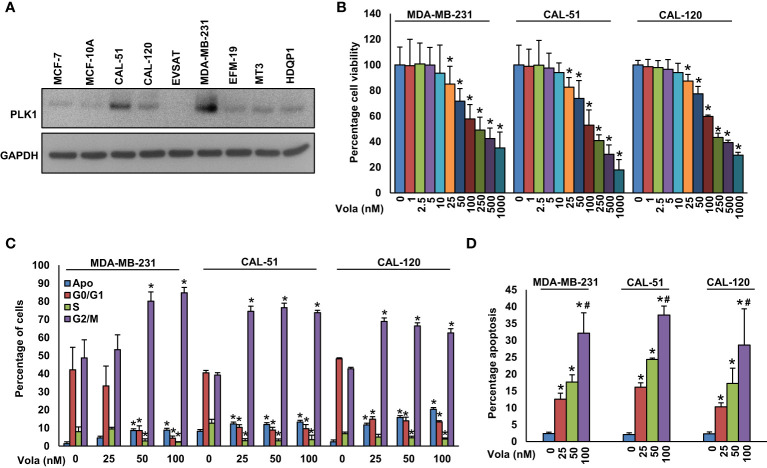
Inhibition of PLK1 impedes TNBC cell growth. **(A)** Basal expression of PLK1 in MCF-10A and BC cells. Proteins were isolated from MCF-10A and BC cells and immunoblotted with PLK1 and GAPDH antibodies (n=3). **(B)** Volasertib inhibits cell viability. TNBC cells (10^4^) were treated with various doses of volasertib for 48 h and MTT assay was performed (n=6). **(C)** Effect of volasertib on cell cycle. The TNBC cells were exposed to different doses of volasertib for 24 hours. Following incubation, cells were analyzed for cell cycle fractions by flow cytometry (n=3). **(D)** Volasertib induces apoptosis in TNBC cell lines. TNBC cells were exposed to different doses of volasertib for 48 h and cells were stained with annexin-V/PI followed by flow cytometry analysis (n=3). * and # indicate statistically significant differences compared to control without treatment and volasertib (50 nM) treatment, respectively with p < 0.05.

We established a notable correlation between PLK1 and TNBC in a large cohort of BC cases. Consequently, we aimed to ascertain whether blocking PLK1 could serve as a viable therapeutic approach to combat the highly aggressive TNBC cells, both *in vitro* and *in vivo*. Initially, we assessed the expression of PLK1 in a group of BC cell lines, as well as the MCF10A cell line, utilizing western blot analysis (**Figure 2A**). This analysis led to the identification of three TNBC cell lines; MDA-MB-231 (*BRCA1* mutant), CAL-51 (*BRCA2* mutant) and CAL-120 (*BRCA* wild type) with high PLK1 expression and we selected these cell lines for further experimentation. Subsequently, we treated these cell lines to varying concentrations of volasertib for a duration of 48 hours and assessed their cell viability. The MTT assay revealed a dose-dependent reduction in cell viability across these cell lines following volasertib treatment (**Figure 2B**).

To delve deeper into the mechanism behind volasertib’s impact on cell growth, we exposed TNBC cells to volasertib (25, 50 and 100 nM) for 24 hours and conducted cell cycle analysis. As anticipated, the 24-hour treatment with volasertib led to a notable decrease in the cell population in the G1 phase of the cell cycle, concomitant with an arrest in the G2/M phase and an increase in apoptosis (**Figure 2C**). Notably, annexin V/PI revealed a remarkable increase in apoptotic cells following 48 hours of volasertib treatment in TNBC cells (**Figure 2D**).

We also showed that knockdown of PLK1 in MDA-MB-231 cells delayed tumor growth *in vivo* as observed by reduced tumor volume (**Supplementary Figures S1A, B**) and weight (**Supplementary Figure S1C**). To further endorse these results, nude mice bearing MDA-MB-231 xenografts were given 10 and 20 mg/kg volasertib injection intraperitoneally for 4 weeks. We noticed only a moderate delay in the tumor growth (**Supplementary Figures S1D–F**) after volasertib treatment, however the mice treated with 20 mg/kg volasertib demonstrated a significant (p<0.05) difference in tumor volume (**Supplementary Figures S1D, E**) and weight (**Supplementary Figure S1F**) compared to vehicle (0.1% DMSO) treated control mice. These results imply that the inhibition of PLK1 holds the potential to reduce the growth of TNBC cells, both *in vitro* and *in vivo*.

### Co-inhibition of PLK1 and PARP synergistically persuaded apoptosis in BC cells

**Figure 3:**
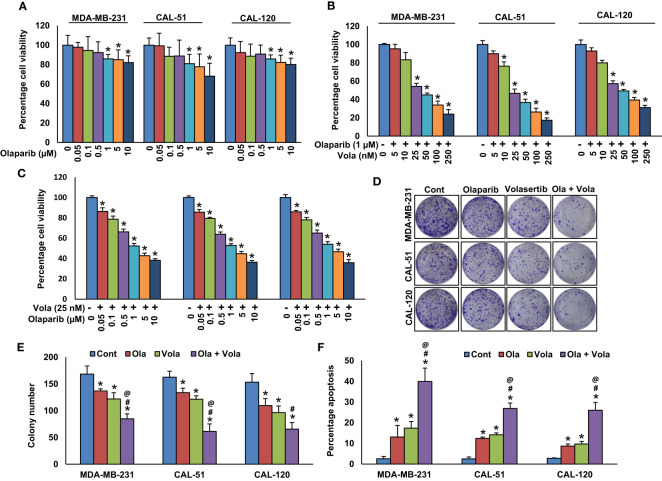
Co-inhibition of PLK1 and PARP synergistically induce apoptosis in BC cells. **(A)** Effect of olaparib on TNBC cell viability. TNBC cells were treated with increasing doses of olaparib for 48 hours. **(B, C)** Synergistic effect of olaparib and volasertib on TNBC cell viability. TNBC cells were treated with indicated doses of olaparib, volasertib and its combination for 48 hours and MTT assay was performed. **(D, E)** Olaparib and volasertib synergistically inhibits clonogenicity. TNBC cells (5 × 10^2^) after olaparib and volasertib treatments were plated into 60 mm diameter dishes and cultured for 10 days then colonies were stained with crystal violet and counted. Data were presented as mean ± SD (n = 3). **(F)** Olaparib and volasertib synergistically induce apoptosis in TNBC cells. TNBC cells were treated with olaparib, volasertib and combination for 48 hours and cells were stained with annexin-V/PI and analyzed by flow cytometry. Data presented in the bar graphs are the mean ± SD (n=3).*, # and @ indicate statistically significant differences compared to control without treatment, olaparib alone and volasertib alone treatment, respectively with p < 0.05.

We established a significant correlation between PLK1 and PARP in our BC cohort. Consequently, we sought to explore whether concurrently targeting PLK1 and PARP with specific inhibitors could hinder the growth of TNBC cells in these cell lines. Our findings demonstrated that olaparib alone showed only a partial growth inhibition in TNBC cells (**Figure 3A**). However, when combining different doses of volasertib with suboptimal doses of olaparib (**Figure 3B**), and vice versa (**Figure 3C**), we observed a synergistic effect that significantly impeded the growth of TNBC cells. By employing calcusyn software (54), we calculated the Combination Index (CI), and the results indicated strong synergism between volasertib at 25 nM and olaparib at 1 μM in three TNBC cell lines. Specifically, in MDA-MB-231 cell line, the CI was 0.220 (**Supplementary Figure S2**), in CAL-51 cell line, it was 0.196 (**Supplementary Figure S3**), and in CAL-120 cell line, it was 0.192 (**Supplementary Figure S4**), further underscoring the potent synergistic effect of this combination. Employing these specific doses, we observed a significant reduction in colony numbers of TNBC cells when treated with the combination of volasertib and olaparib compared to cells treated with either inhibitor alone (**Figures 3D, E**).

To delve into the potential synergy between PLK1 and PARP inhibition on inducing apoptosis, TNBC cells were exposed to volasertib and olaparib individually or in combination for 48 hours. Following this treatment, the cells were subjected to dual staining with annexin V/PI, and subsequent flow cytometry analysis was performed. The treatment with olaparib alone induced 13.09± 6.8% and volasertib alone induced 17.42 ± 3.8% apoptosis in MDA-MB-231 cells, whereas combination of olaparib and volasertib synergistically (p < 0.05) induced 39.94 ± 7.9% apoptosis (**Figure 3F**). We observed almost similar synergistic effect in CAL-51 and CAL-120 cells (**Figure 3F**).

**Figure 4:**
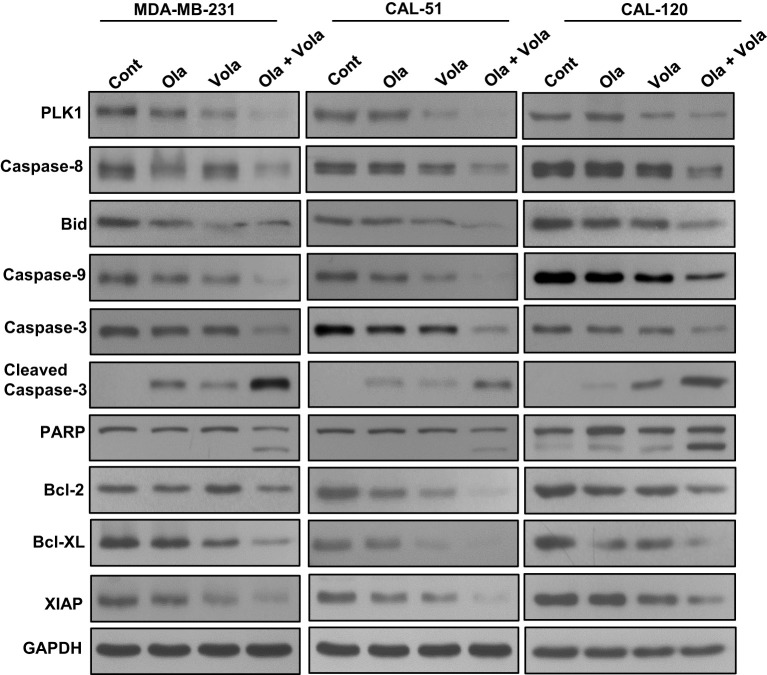
Olaparib and volasertib activate caspase-8 mediated apoptotic signaling pathway in TNBC cells. TNBC cells were treated with indicated doses of olaparib, volasertib and combination for 48 hours. Proteins isolated were subjected to immuno-blotting (n=3).

Previously (25), we showed that olaparib triggers caspase-8 arbitrated extrinsic apoptotic signalling cascade in BC cells. Hence, our aim was to explore whether the treatment with olaparib and volasertib triggers caspase-8 activation and Bid truncation in TNBC cells. Our findings indeed demonstrated caspase-8 activation and Bid truncation following the treatment with olaparib and volasertib in these cells (**Figure 4**).

Additionally, the concurrent administration of olaparib and volasertib elicited the activation and cleavage of Caspase-3, Caspase-9, and PARP in these cell lines (**Figure 4**). Truncated Bid undergoes translocation to the mitochondrial membrane, where it initiates the activation of Bak or Bax while simultaneously deactivating anti-apoptotic proteins, Bcl-2 and Bcl-xl (25). Our research uncovered a reduction in the expression of Bcl-2 and Bcl-Xl subsequent to the concurrent treatment of olaparib and volasertib in these cell lines (**Figure 4**). Furthermore, this combined treatment synergistically reduced the levels of IAPs (inhibitor of apoptosis proteins), notably XIAP, which plays a pivotal role in suppressing apoptosis (**Figure 4**).

### Inhibition of PLK1 attenuates TNBC cell stemness

**Figure 5:**
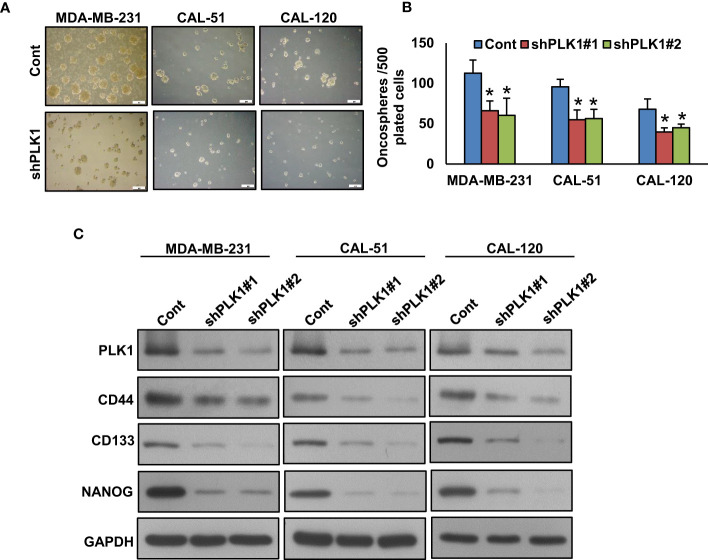
Inhibition of PLK1 reduces the sphere forming ability of TNBC cells. **(A, B)** Knockdown of PLK1 reduce the self-renewal ability of TNBC spheroid cells. TNBC cells were transfected with two different sequence of *PLK1* shRNA’s and the selected clones were cultured in spheroid growth media (scale bar = 1 mm) and spheroids were counted after 10 days. Data were presented as mean ± SD (n = 3) with *p < 0.05 compared to control. **(C)** Knockdown of PLK1 reduces the stemness of spheroids. Proteins were isolated from spheroids and immunoblotted with PLK1, CD44, NANOG, CD133 and GAPDH antibodies (n=3).

**Figure 6:**
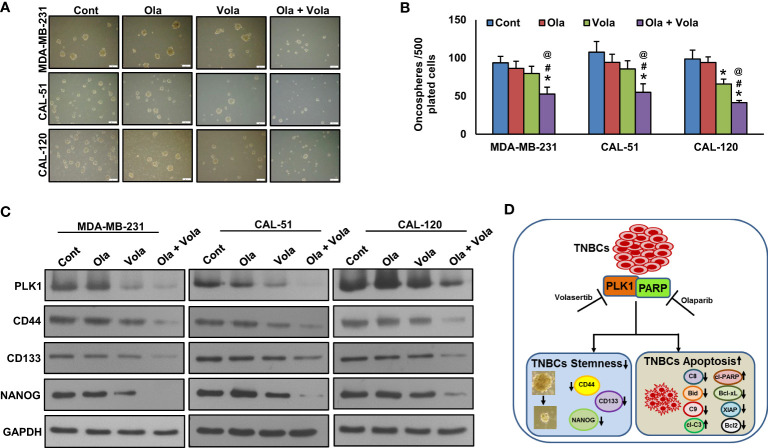
Olaparib and volasertib synergistically reduces spheroid growth. **(A, B)** TNBC cells were incubated with different doses of olaparib, volasertib and combination for 48 hours and allowed to grow in spheroid media (scale bar = 1 mm) and spheroids generated were counted after 10 days. Data were expressed as mean ± SD (n = 3). *, # and @ indicate statistically significant differences compared to control without treatment, olaparib alone and volasertib alone treatment, respectively with p < 0.05. **(C)** Olaparib and volasertib synergistically reduce the stemness. Proteins isolated from spheroids were subjected to immunoblotting (n=3), *p < 0.05. **(D)** Schematic diagram illustrating the mechanism that combined inhibition of PLK1 and PARP in TNBC cells attenuates stemness and induces apoptosis. C8 (Caspase-8), C9 (Caspase-9) and cl-C3 (cleaved Caspase-3).

A recent study has demonstrated that PLK1 plays a significant role in maintaining cancer stem cell properties (55). To explore the role of PLK1 on TNBC stemness maintenance, we established a stable knockdown of PLK1 in these cells and cultured them in spheroid medium. Our results revealed that the knockdown of PLK1 not only reduced the growth of TNBC cell spheroids (**Figures 5A, B**) but also inhibited the expression of key stem cell markers, including CD133, CD44, and NANOG (**Figure 5C**). Additionally, we explored the effect of olaparib, volasertib, and their combination on the stemness of TNBC cells. Our findings demonstrate that the co-treatment of olaparib and volasertib led to a significant reduction in spheroid growth (**Figures 6A, B**) and the stemness characteristics (**Figure 6C**) of the tested TNBC cells.

## Discussion

PLK1, a pivotal controller of mitotic cell division, is a promising prognostic surrogate in BC (56). Additionally, in a previous study, PLK1 was identified among the genes linked to atypical mitotic events (57). It has been suggested that elevated PLK1 expression is correlated with an unfavorable prognosis in BC (58). However, the role PLK1 in BC pathogenesis from Middle Eastern ethnicity remains unexplored.

To explore the role of PLK1 in this specific ethnic group, we examined the expression of PLK1 protein in a large cohort of primary Middle Eastern BC samples using immunohistochemistry. Our study revealed that elevated PLK1 expression was observable in 27% of the cases we analyzed. In a general, high PLK1 expression tends to be associated with more aggressive tumor traits and a higher mitotic score. Interestingly, PLK1 overexpression was enriched in TNBC tumors as compared with luminal BC (43.8% vs 25.4%, p < 0.0001), proposing that PLK1 plays a more important role in Middle Eastern TNBC and functions as an oncogene in TNBC, which is supported in several previous studies (59, 60).

Moreover, elevated PLK1 expression demonstrated a significant association with unfavorable overall survival outcomes in univariant analysis. Our results align with recent research that has demonstrated the prognostic importance of PLK1 in BC, especially in TNBC subtype (59–61). In our cohort, patients experienced significantly worsened survival outcomes when their tumors exhibited co-expression of PLK1 and PARP. This observation raises the possibility that these two genetic abnormalities might interact synergistically, potentially influencing the survival outcomes of BC in this ethnic group.

Cancers with *BRCA1/2* mutations including TNBC and ovarian cancer demonstrate sensitivity to PLK1 inhibitors like onvansertib (62–64). The utilization of PLK1 inhibition as an approach for treating TNBC and *BRCA* defined cancers and the clinical association between PLK1 and PARP in our cohort intrigued us to explore whether combinatorial, sequential inhibition of PLK1 and PARP might be a good therapeutic approach to extend the potential effect of PLK1 inhibitor beyond BRCA deficient BC.

The antitumor effect of volasertib and olaparib alone and its combination was tested in two *BRCA* mutated and one *BRCA* proficient TNBC cell lines. Based on our findings, it appears that PLK1 inhibition reinstates sensitivity to PARPi in all TNBC cell lines, including *BRCA* proficient TNBC cell line, tested. The combination of PLK1 inhibitor (PLKi) and PARPi drastically decreased TNBC cell survival and induced apoptosis.

In summary, our findings indicate that the elevated expression of PLK1 is notably prevalent in TNBC, suggesting its potential utility as a prognostic marker for this aggressive subtype in Middle Eastern BC. *In vitro* data suggest PLK1 inhibition impaired clonogenic potential and increased G2-M arrest and apoptosis in TNBC cell lines. Inhibition of PLK1 overcame PARPi resistance, and its combined inhibition attenuated stemness and induced apoptosis in TNBC cells (**Figure 6D**), which could potentially bolster the clinical development of combination therapy. This combined targeted strategy could potentially broaden the scope of PLK1 inhibition therapy beyond *BRCA*-deficient TNBC in the future.

## Data availability statement

The original contributions presented in the study are included in the article/**Supplementary Material**. Further inquiries can be directed to the corresponding author.

## Ethics statement

Ethical approval was not required for the studies on humans in accordance with the local legislation and institutional requirements because only commercially available established cell lines were used. The animal study was approved by Institutional Review Board of the King Faisal Specialist Hospital and Research Center. The study was conducted in accordance with the local legislation and institutional requirements.

## Author contributions

AS: Conceptualization, Investigation, Project administration, Supervision, Writing – original draft. PP: Data curation, Formal Analysis, Investigation, Software, Validation, Visualization, Writing – original draft, Writing – review & editing. DP: Data curation, Investigation, Validation, Writing – review & editing. SP: Data curation, Investigation, Validation, Writing – review & editing. KA: Data curation, Writing – review & editing. ST: Data curation, Writing – review & editing. RD: Data curation, Writing – review & editing. RB: Data curation, Writing – review & editing. OA: Resources, Writing – review & editing. FA-D: Resources, Writing – review & editing. KA-K: Conceptualization, Funding acquisition, Investigation, Project administration, Resources, Supervision, Writing – original draft, Writing – review & editing.
