# Supplementary material for: PLK1 and PARP positively correlate in Middle Eastern breast cancer and their combined inhibition overcomes PARP inhibitor resistance in triple negative breast cancer
Source: Front Oncol. 2024 Jan 3;13:1286585. doi: 10.3389/fonc.2023.1286585 (PMC10791948; doi:10.3389/fonc.2023.1286585)
Supplement: Supplementary file 1 [file DataSheet_1.pdf]

Supplementary materials

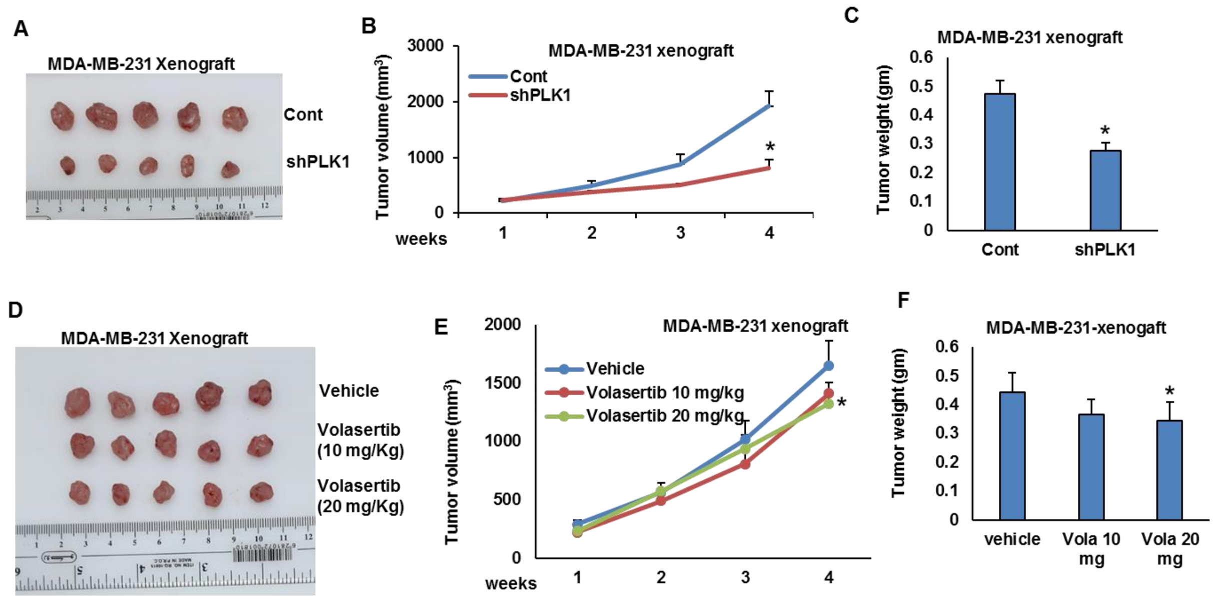

Supplementary Figure 1. Inhibition of PLK1 delays TNBC tumor growth in vivo. (A-C) Knockdown of PLK1 in MDA-MB-231 cells delayed tumor growth in vivo. PLK1 knockdown MDA-MB-231 cells and their control cells were subcutaneously injected into the flanks of 6-week old nude mice (4×10^6 cells per mouse). (B) The volume of each tumor was measured every week. The average (n = 5) tumor volume (± SD) in each group of mice was calculated, *$p < 0.05$. (C) After four weeks, mice were sacrificed and mean tumor weight (± SD) was calculated in each group, *$p < 0.05$. (D-F) Volasertib inhibits TNBC tumor growth in vivo. MDA-MB-231 cells were subcutaneously injected into the flanks of 6-week old nude mice (4×10^6 cells per mouse). After tumors grew to about 100 mm^3, mice were treated intraperitoneally with indicated dose of volasertib, twice a week for 30 days. (D) Representative tumor images of each group of mice. (E) The volume of each tumor was measured every week. The average (n = 5) tumor volume (± SD) in each group of mice was calculated, *$p < 0.05$. (F) After four weeks treatment, mice were sacrificed and mean tumor weight (± SD) was calculated in each group, *$p < 0.05$.

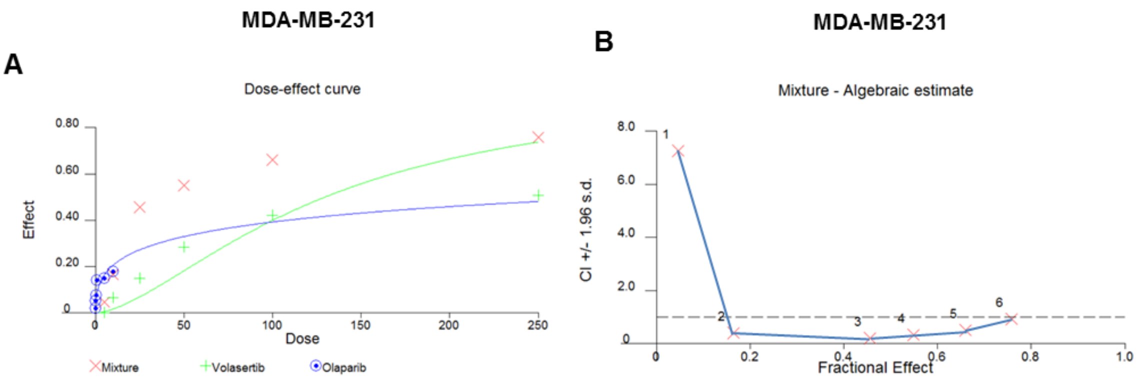

C

Combination index (CI) for experimental values			
**Volasertib (nM)**	**Olaparib (μM)**	**Fa**	**CI**
**5**	**1**	0.046	7.261
**10**	**1**	0.165	0.416
**25**	**1**	0.457	0.220
**50**	**1**	0.549	0.342
**100**	**1**	0.660	0.506
**250**	**1**	0.759	0.930

Supplementary Figure 2. Synergistic inhibition of cell viability by olaparib and volasertib in MDA-MB-231 cells. MDA-MB-231 cells were treated with various combinations of volasertib and olaparib for 48 h and dose effect (A) and Fractional effect (B) graphs were generated using Calcusyn software. (C) Combination Index (CI) were calculated using Chou and Talalay method.

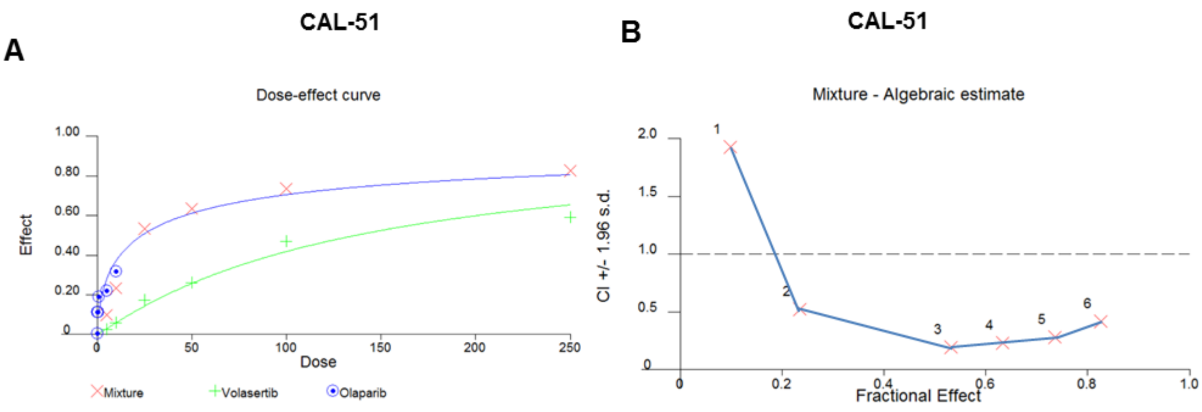

C

Combination index (CI) for experimental values			
**Volasertib (nM)**	**Olaparib (µM)**	**Fa**	**CI**
**5**	**1**	0.099	1.927
**10**	**1**	0.235	0.523
**25**	**1**	0.532	0.196
**50**	**1**	0.633	0.235
**100**	**1**	0.735	0.285
**250**	**1**	0.825	0.422

Supplementary Figure 3. Synergistic inhibition of cell viability by olaparib and volasertib in CAL-51 cells. CAL-51 cells were treated with various combinations of volasertib and olaparib for 48 h and dose effect (A) and Fractional effect (B) graphs were generated using Calcusyn software. (C) Combination Index (CI) were calculated using Chou and Talalay method.

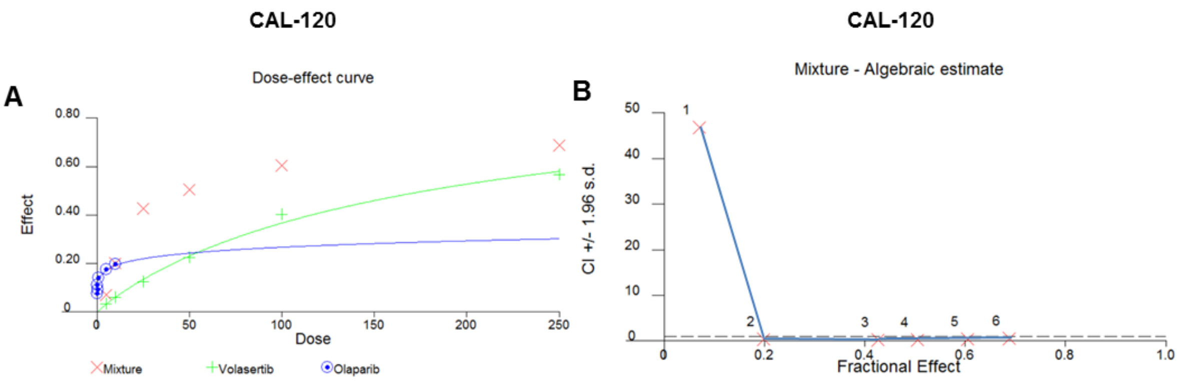

C

Combination index (CI) for experimental values			
**Volasertib (nM)**	**Olaparib (μM)**	**Fa**	**CI**
**5**	**1**	0.070	46.74
**10**	**1**	0.199	0.321
**25**	**1**	0.427	0.192
**50**	**1**	0.506	0.274
**100**	**1**	0.605	0.358
**250**	**1**	0.689	0.607

Supplementary Figure 4. Synergistic inhibition of cell viability by olaparib and volasertib in CAL-120 cells. Cal-120 cells were treated with various combinations of volasertib and olaparib for 48 h and dose effect (A) and Fractional effect (B) graphs were generated using Calcusyn software. (C) Combination Index (CI) were calculated using Chou and Talalay method.
